# Concerns regarding the safety and efficacy of ES-Cu-Captisol for Menkes disease. Reply.

**DOI:** 10.1172/JCI202684

**Published:** 2026-02-16

**Authors:** Vishal M. Gohil, Michael J. Petris, Francesc Palau

**Affiliations:** 1 Department of Biochemistry & Biophysics, Texas A&M University, College Station, Texas, USA.; 2 Departments of Ophthalmology and Biochemistry, University of Missouri, Columbia, Missouri, USA.; 3 Center for Genomic Sciences in Medicine, Sant Joan de Déu Hospital and Research Institute, and CIBERER, University of Barcelona, Barcelona, Spain.

**Keywords:** Clinical Research, Genetics, Drug therapy, Genetic diseases, Neurological disorders

**The authors reply:** We appreciate the opportunity to respond to the Letter by Kaler (1) regarding our recent report on adjunctive elesclomol-copper (ES-Cu) therapy in 2 infants with Menkes disease (2). We welcome the scrutiny and take this opportunity to clarify how the contribution of ES-Cu was distinguished from that of copper histidinate (CuHis), to address concerns about dosing and mechanism, and to further contextualize safety and anticipated therapeutic benefits.

In our study, the therapeutic effect of ES-Cu was evaluated using two complementary approaches. First, named patient 1 (NP#1) served as his own internal control. NP#1 received daily subcutaneous CuHis beginning on day 5 of life, yet by 20 months of treatment his neurodevelopmental scores across cognitive, expressive language, gross motor, and fine motor domains remained below the fifth percentile. Subcutaneous ES-Cu was added at that time as an adjunct to CuHis. Within 2 months, NP#1 exhibited marked and sustained gains across all domains, with neurodevelopmental scores increasing by multiple centiles each month. Within 10 months, NP#1 achieved or surpassed the fiftieth percentile in most domains (2). Because CuHis dosing was maintained, and because the comparison reflects trajectories both before and after ES-Cu treatment, these findings strongly suggest that the addition of ES-Cu contributed substantially to the neurological improvements.

Second, a comparison of NP#1 and NP#2 provides further evidence of the benefit of ES-Cu treatment compared with that of CuHis alone. Both infants began CuHis during the first week of life; however, ES-Cu was initiated at 2 months (corrected age) in NP#2 versus 20 months in NP#1. NP#2 achieved all 8 major gross motor milestones as much as 9 months sooner depending on the milestone (2). These between-patient comparisons, aligned with the marked improvement of NP#1 after ES-Cu initiation, support the conclusion that ES-Cu provides additional benefit beyond CuHis alone.

Kaler argues against the therapeutic efficacy of ES-Cu because it represented a low fraction of total weekly copper given to patients relative to CuHis (7.7% in NP#1; 4.46% in NP#2) (1). However, this argument rests on an erroneous assumption that both molecules exhibit equivalent potency. It has been well documented that ES-Cu and CuHis fundamentally differ in physicochemical properties and copper release mechanisms. For example, the lipophilic ES-Cu complex is 5,000-fold more potent than hydrophilic CuHis at delivering copper to cytochrome *c* oxidase due to its ability to cross lipid bilayers and release copper within the mitochondrial matrix (3). Moreover, in a mouse model of Menkes disease, ES-Cu or CuHis administered at equivalent copper doses produces dramatically different outcomes. Only ES-Cu elevated brain copper levels, restored cuproenzyme, and rescued neuropathology and mortality (4). Thus, the absolute copper mass contributed by ES-Cu is not an appropriate metric for predicting therapeutic impact.

We agree with Kaler that injection-site inflammation is an important adverse effect to monitor. In our experience, these localized reactions occur within 24 hours of weekly ES-Cu dosing, resolve quickly, and are manageable with topical or oral antiinflammatory medications. The absence of systemic toxicity and the clinical gains achieved suggest a favorable risk-benefit ratio when used under close supervision and with mitigation strategies (5). In a prior publication, Kaler highlight’s the limitations of CuHis treatment stating, “the relatively high rate of under-three mortality for this orphan disease regardless of when copper injection treatment is initiated also indicates the need for supplemental therapeutic approaches” (6). We view localized and manageable injection-site reactions as acceptable when weighed against an otherwise dismal prognosis.

Our findings do not unequivocally establish that ES-Cu alone is solely responsible for the observed outcomes. As stated explicitly in Godoy-Molina et al., “our results with ES-Cu, combined with CuHis, provide preliminary evidence of the safety and efficacy of this promising treatment regimen” (2). Our intent was not to diminish the established value of CuHis, but rather to highlight the clinical, biochemical, and mechanistic benefits that ES-Cu offers beyond standard copper replacement, particularly in infants with severe *ATP7A* loss-of-function variants. We respectfully submit that the remarkable improvements in neurodevelopmental milestones observed in our cases upon the addition of ES-Cu therapy suggests a substantive therapeutic advance worthy of further study.

## Acknowledgments

The authors thank the patients and their families for their participation in this study, as well as the Menkes International Association (MIa) and the Amigos de Nono Foundation for their support.

## Version 1 02/16/2026

Electronic publication

## References

[1] Kaler S (2026). Concerns regarding the safety and efficacy of ES-Cu-Captisol for Menkes disease. J Clin Invest.

[2] Godoy-Molina E (2025). Elesclomol-copper therapy improves neurodevelopment in two children with Menkes disease. J Clin Invest.

[3] Zulkifli M (2023). FDX1-dependent and independent mechanisms of elesclomol-mediated intracellular copper delivery. Proc Natl Acad Sci U S A.

[4] Guthrie LM (2020). Elesclomol alleviates Menkes pathology and mortality by escorting Cu to cuproenzymes in mice. Science.

[5] Zulkifli M (2025). Elesclomol rescues mitochondrial copper deficiency in disease models without triggering cuproptosis. J Pharmacol Exp Ther.

[6] Kaler SG (2014). Neurodevelopment and brain growth in classic Menkes disease is influenced by age and symptomatology at initiation of copper treatment. J Trace Elem Med Biol.

